# Risk Factors of Mortality in Severely-malnourished Children Hospitalized with Diarrhoea

**DOI:** 10.3329/jhpn.v29i3.7870

**Published:** 2011-06

**Authors:** S.K. Roy, Maaike Buis, Renee Weersma, Wajiha Khatun, S. Chowdhury, Afroza Begum, Debjani Sarker, Saima Kamal Thakur, Mansura Khanam

**Affiliations:** ^1^ICDDR,B, GPO Box 128, Dhaka 1000, Bangladesh; ^2^University of Amsterdam, Amsterdam, The Netherlands

**Keywords:** Bronchopneumonia, Case-control studies, Child nutritional disorders, Diarrhoea, Hypoglycaemia, Leukocytosis, Marasmic-kwashiorkor, Mortality, Risk factors, Bangladesh

## Abstract

This case-control study was conducted in the Dhaka Hospital of ICDDR,B to identify the risk factors of mortality in severely-malnourished children hospitalized with diarrhoea. One hundred and three severely-malnourished children (weight-for-age <60% of median of the National Center for Health Statistics standard) who died during hospitalization were compared with another 103 severely-malnourished children who survived. These children were aged less than three years and admitted to the hospital during 1997. On admission, characteristics of the fatal cases and non-fatal controls were comparable, except for age. The median age of the cases and controls were six and eight months respectively (p=0.05). Patients with low pulse rate or imperceptible pulse had three times the odds of death compared to the control group (p<0.01). The presence of clinical septicaemia and clinical severe anaemia had 11.7 and 4.2 times the odds of death respectively (p<0.01). Patients with leukocytosis (>15,000/cm^3^) had 2.5 times the odds of death (p<0.01). Using logistic regression, clinical septicaemia [adjusted odds ratio (AOR)=8.8, confidence interval (CI) 3.7-21.1, p=0.01], hypothermia (AOR=3.5, CI 1.3-9.4, p<0.01), and bronchopneumonia (AOR=3.0, CI 1.2-7.3, p<0.01) were identified as the significant risk factors of mortality. Severely-malnourished children (n=129) with leukocytosis, imperceptible pulse, pneumonia, septicaemia, and hypothermia had a high risk of mortality. The identified risk factors can be used as a prognostic guide for patients with diarrhoea and severe malnutrition.

## INTRODUCTION

Diarrhoea is one of the most important causes of death in the world. Globally, more than 10 million children die each year, of whom about 1.5 million die from diarrhoea (1,2).

Diarrhoea and malnutrition are common in young children in developing countries, and a reciprocal relationship has been postulated between diarrhoea and malnutrition. Malnutrition is a determining factor for duration of diarrhoea but not incidence (3).

In Bangladesh, diarrhoea is a major cause of child mortality. According to the United Nations Children's Fund (2004), 36,000 children aged less than five years (under-five children) die due to diarrhoea each year (4). According to the Bangladesh Demographic and Health Survey 2007, 10% of under-five children had diarrhoea in two weeks before the survey (5).

Deaths among children hospitalized with diarrhoea in Bangladesh are significantly higher among those who had altered consciousness, hypoglycaemia, septicaemia, paralytic ileus, toxic colitis, necrotizing enterocolitis, haemolytic-uraemic syndrome, invasive or persistent diarrhoea, dehydration, electrolyte imbalances, and malnutrition (6). Female babies experienced a two-fold higher risk of death than male babies. Female babies with severe infection were less-frequently hospitalized compared to male babies (7).

Malnutrition, a direct and associated cause of infant mortality, is a major contributor to the burden of disease in developing countries. Chen *et al*. reported that severely-malnourished under-two children had four times higher risk of death compared to well-nourished children in Bangladesh (8). Results of a study in 1983 showed that severely-malnourished children discharged after recovery from diarrhoea had 14 times higher risk of death than their well-nourished counterparts, with the highest mortality among two-year old children (9). Results of a recent study showed that management of severe acute malnutrition following the guidelines of the World Health Organization (WHO) reduced the case-fatality rate of children by 55% (risk ratio=0.45, 95% confidence interval (CI) 0.32-0.62) (10).

The aim of the present study was to determine the specific risk factors of mortality associated with diarrhoea among severely-malnourished children aged less than three years hospitalized for diarrhoea.

## MATERIALS AND METHODS

### Study design

A case-control study was conducted among 206 severely-malnourished subjects—103 patients who died from acute diarrhoea were taken as cases and 103 patients who survived were selected as controls—during January-December 1997 admitted to the Dhaka Hospital of ICDDR,B.

### Definition of case and control

Cases and controls were selected from among severely-malnourished children with acute diarrhoea aged less than three years. Of those children who died were included as cases and those who survived were randomly selected as controls. Severe malnutrition was defined as a weight-for-age (W/A) less than 60% according to the National Centre for Health Statistics (NCHS) standard, or presence of oedema. Acute diarrhoea was defined as the passage of three or more loose or liquid stools per day (11).

### Definition of diarrhoea

According to the WHO, diarrhoea is defined as the passage of unusually loose or watery stools, usually at least three times in a 24-hour period. In acute diarrhoea, watery stool lasts for several hours or days, and the main danger is dehydration. Diarrhoea that lasts for 14 days or longer is called persistent or chronic diarrhoea. In persistent diarrhoea, the main danger is malnutrition; serious non-intestinal infection and dehydration may also be present (11).

### Study population

The Dhaka Hospital of ICDDR,B is a specialized diarrhoeal disease hospital that has been functioning since 1963 for the treatment of diarrhoeal diseases in Bangladesh. Currently, the hospital cares for more than 120,000 patients each year. In 1997, 114,987 patients attended the Dhaka Hospital, of which 61,911 (53.84%) were admitted to the short-stay ward and 5,531 (4.81%) to the longer-stay ward. Of them, 292 (0.25%) died. The cases were selected from these dead patients, and controls were selected from those who survived.

### Sample-size

To calculate the sample-size, approximately 15% of the controls would be exposed to the risk factors assumed. An odds ratio of 3.0 was thought to be worth detecting with 80% power and 5% significance. Taking one control per case, the sample-size calculated was 85 for cases and the same number for control. However, all 103 severely-malnourished children who died during 1997 and the same number of controls were included to cover any missing information.

### Selection of subjects

All the cases and controls were severely malnourished defined by weight-for-age <60% of median of the NCHS standard. For each case, a matchedcontrol was identified, who was admitted during the same period (within 3 days) and was of the same sex but recovered from illness. Patients suffering from any chronic illnesses or those who were referred to other hospitals or left the hospital against advice of the physicians or absconded from the hospital were not included.

### Hospital treatment

Patients with some dehydration were treated with oral rehydration salts solution and those with severe dehydration received intravenous fluid according to the guidelines of WHO (11). Patients with complications, such as electrolyte imbalance, hypoglycaemia, lower respiratory tract infection, severe protein-energy malnutrition or septicaemia were admitted to the longer-stay ward. They comprised about 5% of the total numbers of patients attending the hospital.

A routine check-up was performed on all patients admitted to the longer-stay ward. A physician assessed the dehydration status and recorded all clinical signs and major complications, made diagnosis, and advised for investigations and treatment. A rectal swab or stool specimen was obtained for culture of *Shigella, Salmonella,* and *Vibrio cholerae*. Blood samples were cultured for suspected septicaemia. If symptoms and signs of pneumonia or other lung diseases were suspected, x-ray of the chest was performed.

All the severely-malnourished children were treated in the general ward and in the Special Care Unit. Initial treatments of life-threatening conditions, such as infection, dehydration, hypoglycaemia, hypothermia, specific deficiencies, and metabolic abnormalities, were given. The key elements of management included slowing rehydration with an emphasis on oral rehydration, early start of feeding, routine supplementation of micronutrients (zinc, magnesium, multivitamins, vitamin A, and folic acid), broad-spectrum antibiotic therapy (ampicillin and gentamicin for children without pneumonia and chloramphenicol for children with pneumonia (12). A normal hospital diet (Table 1) was given to children with acute diarrhoea (13).

**Table 1.** Composition of low lactose milk-based diet for acute diarrhoea patients (per litre) (16)

Powdered milk	35 g
Rice-powder	30 g
Sugar	25 g
Oil	20 mL
Sodium chloride	1 g
Water (up to)	1 L
Energy	2,800 kJ (670 kcal)
Protein	17 g
Elemental zinc	1.4 mg

### Information from records of patients

A pre-coded questionnaire was used for recording relevant information from the hospital-records. The questionnaire was pretested before obtaining information from the hospital-records. Data on degree of malnutrition clinically categorized as kwashiorkor, marasmic-kwashiorkor and marasmus, presence of oedema, duration of diarrhoea and vomiting before admission, diarrhoeal pathogen, presence of severe anaemia (based on clinical examination), clinically-diagnosed pneumonia, septicaemia, respiratory distress and shock, serum electrolytes, blood glucose level on admission, complete white cell count, signs of xerophthalmia, fever, socioeconomic condition, immunization, and feeding status, were collected from the hospital-records.

### Statistical analysis

Data were entered into the microcomputer and checked using the SPSS software (version 11.5). Des-criptive statistics were used for the key variables. Comparison of categorical variables between the cases and the controls was made using the chi-square test. Fisher's, exact test was used where the expected count was less than five. Comparison of continuous variables was done with the independent sample *t*-test for normally-distributed data or with the Mann-Whitney U-test for non-normal distribution. Two-sided significant test was used. To identify the factors independently associated with an increased risk of death, multiple logistic regression analysis was carried out. In a backward stepwise regression, all non-significant variables were eliminated until a final model with significant p values and odds ratios was reached. The Epi Info software **(**version 3.4.3**)** was used for calculating the degree of malnutrition.

## RESULTS

### Results of univariate analysis

Results of the laboratory tests, including serum sodium, serum potassium, serum chloride, serum TCO_2_, total white cell count, haematocrit, and bands, were not available for all the cases and controls. Other variables, such as weight-for-height (W/H), duration of diarrhoea, duration of vomiting, degree of dehydration, socioeconomic condition, blood glucose level, presence of pathogens during diarrhoea, and breastfeeding, were also not available for all the patients.

Table 2 shows the results of comparison of sociodemographic characteristics, feeding, and nutritional status of fatal cases and non-fatal controls on admission.

**Table 2.** Comparison of characteristics on admission of severely-malnourished patients who died and who survived

Characteristics	Cases (n=103)		Controls (n=103)		p value
	No.	%	No.	%	
Sex			
Male	54 52	54 52	0.84
Female	49 48	49 48	
Age (months)			
0-23 [median (range)]	6 (0.5-30)	8 (0.75-30)	0.05
<1	8 8	5 5	
1-6	48 47	36 35	
7-13	26 25	35 34	
14-24	17 17	26 25	
>24	4 4	1 1	
Duration (days) of diarrhoea beforeadmission [median (range)]	5 (0.25-60)	5 (0.5-30)	0.72
Immunized	54 52	66 64	0.55
Income (Tk/month)*			
<1,000	32 33	31 32	
1,001-1,500	2 2	9 9	
1,501-3,000	41 43	32 33	
3,001-4,000	8 8	9 9	
>4,000	13 14	16 17	
Breastfeeding			
Exclusive	17 17	17 17	1.05
Partial	51 50	60 60	0.25
Never breastfed	34 33	23 23	

*Tk 46=US$ 1 as in 1997

The cases and controls were comparable with res-pect to all the characteristics, except for age. The age of the fatal cases was significantly (p=0.05) less by two months. Sex distribution of the cases was similar to that of the controls (p=0.84).

Table 3 shows the results of comparison of clinical characteristics between severely-malnourished patients who died and who survived. The severely-malnourished patients with imperceptible pulse/low pulse rate (<90) had three times the odds of death (p<0.01). The presence of hypothermia showed 4.8 times the odds of mortality (p<0.01). The severely-malnourished children with clinical septicaemia had a very high risk (11.7 times) of mortality (p<0.01). Patients with fatal outcome were 13.3 times more likely to suffer from more than two complications (p=0.01). The presence of severe anaemia and bronchopneumonia led to an increased risk of death by 4.2 and 2.14 folds respectively (p<0.01).

**Table 3.** Comparison of clinical characteristics on admission in severely malnourished patients who died and who survived

Clinical characteristics	Case (n=103)		Control (n=103)		Odds ratio 95%CI	pvalue
	No.	%	No.	%		
Low/imperceptible pulse rate (<90)	52 51	26 25	3.0 (1.6-5.7)	0.01
Hypothermia (<36 °C)	40 39	12 12	4.8 (2.2-10.6)	0.01
Clinical septicaemia	83 81	27 26	11.7 (5.8-23.9)	0.01
Some dehydration	52 51	37 36		0.35
Severe dehydration	13 13	8 8		0.25
Marasmic-kwashiorkor*	21 20	13 13	1.8 (0.8-4.0)	0.14
Marasmus*	76 74	90 87	0.4 (0.2-0.9)	0.14
Clinical severe anaemia	15 15	4 4	4.2 (1.6-15.6)	0.01
Bronchopneumonia	66 64	48 47	2.14 (1.1-3.9)	0.01
Respiratory distress	3 3	9 9	0.3 (0.07-1.3)	0.01
More than two complications	12 12	1 1	13.3 (3.0-82.7)	0.01
Duration (days) of hospitalization [median (range)]	1.5 (0.25-19.5)	5 (0.5-44)	-	0.01

*% of NCHS median references;

CI=Confidence interval;

NCHS=National Center for Health Statistics

The severely-malnourished children with diarrhoea combined with acidosis, hypoglycaemia, and leukocytosis (>15,000) were significantly prone to a higher risk of mortality (Table 4). The presence of acidosis had 1.8 times the odds of death (p=0.03). Hypoglycaemia and leukocytosis showed 3.8 and 2.5 times the odds of death respectively (p=0.01).

**Table 4.** Comparison of serum electrolytes, blood biochemistry, and infection present on admission in severely-malnourished children who died and who survived

Characteristics	Cases		Controls		Odds ratio (95 % CI)	p value
	No.	%	No.	%		
Serum electrolytes	(n=102)*	(n=91)*		
Hyponatraemia (<130 mmol/L)	44 43	27 30	1.8 (0.9-3.4)	0.06
Hypernatraemia(>150 mmol/L)	10 10	5 6	1.9 (0.6-6.6)	0.27
Hypokalaemia (<3.5)	60 59	55 60	0.9 (0.5-1.73)	0.89
Hyperkalaemia (>5.5)	12 12	6 7	1.9 (0.6-6.0)	0.30
Chloride (mmol/L) <97	17 17	16 18	0.9 (0.4-2.1)	0.88
Chloride (mmol/L) >106	58 57	49 54	1.1 (0.6-2.1)	0.67
Acidosis (TCO_2_ <14)	71 70	51 57	1.8 (1.0-3.4)	0.03
Alkalosis (TCO_2_ >18)	17 17	17 19	0.87 (0.4-2.0)	0.72
Blood glucose (mmol/L)	(n=98)	(n=81)		
Hypoglycaemia (<3.0)	30 31	9 11	3.8 (1.5-9.8)	0.01
Hyperglycaemia (>6.5)	37 38	23 28	1.6 (0.8-3.2)	0.20
Total WBC count	(n=65)	(n=63)		
Leukopaenia (<5,000)	4 6	3 5	1.3 (0.2-7.8)	0.74
Leukocytosis (>15,000)	34 52	19 30	2.5 (1.2-5.6)	0.01
Haematocrit	(n=66)	(n=63)		
Anaemia <36	44 68	48 76	0.63 (0.3-1.5)	0.23
Pathogen in diarrhoea	(n=79)	(n=99)		
No organism	49 62	48 49	1.7 (0.9-3.3)	
Enteric pathogen	30 38	51 51	0.58 (0.3-1.1)	0.07
Pathogen in blood	(n=103)	(n=103)		
Yes	17 17	7 7	2.71 (1.0-7.6)	0.03

*Data not shown for all children;

CI=Confidence interval;

WBC=White blood cell

### Results of multivariate analysis

The following variables were entered in the model; clinical septicaemia, hypothermia (<36 ^0^C), hypoglycaemia (glucose <3.0 mmol/L), clinical pneumonia, pathogens in stool, pathogen in blood, and age <6 months), and presence of pedal oedema. Table 4 shows the crude and adjusted odds ratios (ORs) with 95% confidence interval and the crude and adjusted p values of all the variables associated with death. The results of multivariate analysis showed that clinical septicaemia, hypothermia, and bronchopneumonia were significantly associated with death.

Patients with fatal outcome were 8.8 times more likely to have clinical septicaemia (p=0.001) and 3.5 times more likely to have hypothermia (p=0.01). The presence of bronchopneumonia had three times the odds of mortality (Table 5).

**Table 5.** Logistic regression model for risk factors of death in severely-malnourished children

Factor	Crude odds ratio (95% CI)	p value	Adjusted odds ratio (95% CI)	Adjusted p value
Clinical septicaemia	11.7 (6.1-2.5)	0.001	8.8 (3.7-21.1)	0.01
Hypothermia (<36 °C)	4.8 (2.3-9.9)	0.001	3.5 (1.3-9.4)	0.01
Hypoglycaemia (<3.0 mmol/L)	3.5 (1.6-8.0)	0.002	2.4 (0.8-7.2)	0.11
Bronchopneumonia	2.1 (1.2-3.8)	0.01	3.0 (1.2-7.3)	0.01
Pathogen in blood	2.7 (1.1-6.9)	0.04	1.8 (0.5-6.6)	0.36
Pathogen in stool	0.6 (0.3-1.1)	0.07	0.8 (0.3-1.8)	0.53
Age (<6 months)	1.8 (1.0-3.1)	0.04	1.9 (0.8-4.6)	0.15
Malnutrition (kw and mk)	2.5 (1.2-5.1)	0.02	2.8 (0.9-8.7)	0.07

CI=Confidence interval;

kw=Kwashiokor;

mk=Marasmic-kwashiorkor

## DISCUSSION

The poor nutritional status of a child is a major risk factor of mortality (7-9); in particular, it has been shown in other studies among hospitalized and discharged patients (14). The relationship between diarrhoeal deaths and associated conditions has been described in earlier studies (5,14). The present study identified the risk factors of mortality among children with diarrhoea who were severely malnourished and hospitalized. In the study, hypothermia, clinical septicaemia, and bronchopneumonia were identified as the major risk factors of mortality among the severely-malnourished children with diarrhoea.

The strength of this study is that it was a well-defined matched case-control study where the cases and controls were selected from among severely-malnourished children with diarrhoea. However, the limitation of the study is that the data used in the study were not representative of mortality among all severely-malnourished children with diarrhoea in Bangladesh. The Dhaka Hospital is a specialized diarrhoeal treatment facility, and the most complicated cases of diarrhoea are admitted to the inpatient department of this hospital. The patients admitted are not representative for those who are managed in the community. This information was sometimes inadequately recorded because the immediate caretakers were not always attending the child. Some variables were subjectively assessed, such as clinically-diagnosed septicaemia or clinically-diagnosed severe anaemia. Hypothermia has been reported in children with diarrhoea and showed 5.7 times the odds of mortality (15), which is similar to the figure of the present study.

Clinical septicaemia is one of the major causes of death diagnosed by a few clinical signs, such as shock and signs of dehydration in these severely-malnourished children and might be related to the pathogen causing diarrhoea. Children with septicaemia and pneumonia had a higher risk of death. Islam *et al*. found that patients diagnosed to have septicaemia had twice the risk of mortality (8).

The results of our study showed that low pulse rate (<90) or imperceptible pulse and bronchopneumonia were important causes of death. A study found that the presence of low pulse rate (<90) or imperceptible pulse and bronchopneumonia led to the increased risk of death in children by 3.9 and 2.8 times respectively (15).

In previous studies, acidosis had three times the odds of death in severely-malnourished children (16,14). Our study found that acidosis had 1.8 times the odds of death.

In conclusion, this study found that septicaemia, pneumonia, and hypothermia were high risk factors of death among diarrhoeal children with severe malnutrition. However, this fatality could be prevented by early use of antibiotics and supportive care.

## ACKNOWLEDGEMENTS

The study was conducted with financial support from ICDDR,B. Current donors providing unres-tricted support include: Australian Agency for International Development (AusAID), Government of the People's Republic of Bangladesh, Canadian International Development Agency (CIDA), Swedish International Development Cooperation Agency (Sida), and Department for International Development, UK (DFID). The authors gratefully acknowledge these donors for their support and commitment to ICDDR,B's research efforts. The authors also gratefully acknowledge the parents of patients for participation in the study.
